# The Impact of COVID-19 on the Diagnosis and Treatment of Lung Cancer at a Canadian Academic Center: A Retrospective Chart Review

**DOI:** 10.3390/curroncol28060360

**Published:** 2021-10-20

**Authors:** Goulnar Kasymjanova, Aksa Anwar, Victor Cohen, Khalil Sultanem, Carmela Pepe, Lama Sakr, Jennifer Friedmann, Jason S. Agulnik

**Affiliations:** 1 Peter Brojde Lung Cancer Center, Jewish General Hospital, McGill University, Montreal, QC H3T1E2, Canada; carmela.pepe@mcgill.ca (C.P.); lsakr@jgh.mcgill.ca (L.S.); jagulnik@jgh.mcgill.ca (J.S.A.); 2 Department of Oncology, McGill University, Montreal, QC H3T1E2, Canada; aksa.anwar@mail.mcgill.ca; 3 Segal Cancer Centre, Medical Oncology, Jewish General Hospital, McGill University, Montreal, QC H3T1E2, Canada; vcohen@jgh.mcgill.ca (V.C.); Jennifer.Friedmann.med@ssss.gouv.qc.ca (J.F.); 4 Segal Cancer Centre, Radiation Oncology, Jewish General Hospital, McGill University, Montreal, QC H3T1E2, Canada; khalil.sultanem@mcgill.ca

**Keywords:** lung cancer, COVID-19, diagnosis and treatment pattern

## Abstract

The large burden of COVID-19 on health care systems worldwide has raised concerns among medical oncologists about the impact of COVID-19 on the diagnosis and treatment of lung cancer patients. In this retrospective cohort study, we investigated the impact of COVID-19 on lung cancer diagnosis and treatment before and during the COVID-19 era. New lung cancer diagnoses decreased by 34.7% during the pandemic with slightly more advanced stages of disease, there was a significant increase in the utilization of radiosurgery as the first definitive treatment, and a decrease in both systemic treatment as well as surgery compared to the pre-COVID-19 era. There was no significant delay in starting chemotherapy and radiation treatment during the pandemic compared to pre-COVID-19 time. However, we observed a delay to lung cancer surgery during the pandemic time. COVID-19 seems to have had a major impact at our lung cancer center on the diagnoses and treatment patterns of lung cancer patients. Many oncologists fear that they will see an increase in newly diagnosed lung cancer patients in the coming year. This study is still ongoing and further data will be collected and analyzed to better understand the total impact of the COVID-19 pandemic on our lung cancer patient population.

## 1. Introduction

Globally, the coronavirus disease 2019 (COVID-19) has slowed down clinical activities, including cancer care, in order to follow public health directives. A cross-sectional study conducted in 356 centers across 54 countries found that 88% of centers had a hard time delivering care to patients, due to the large burden on the health care system, lack of protective equipment, decline in number of healthcare personnel working, and low access to medications [1]. Another recent study reported that due to the COVID-19 pandemic, screening of cancers declined drastically, 85% for breast, 75% for colon, 74% for prostate, and 56% for lung cancer [2]. This may potentially lead to a delayed diagnosis and more advanced stage patients. This finding is consistent with the report issued in January 2021 by the Quebec Ministry of Health stating that a decrease in the number of new cancer diagnoses is expected, given the decline in medical consultations and various services (screening examinations, tests confirming cancer diagnosis, and tumor tissue sent to pathology) [3]. Garassino et al. reported increased mortality in patients with thoracic malignancy who were infected with COVID-19 [4]. 

The large burden of COVID-19 on health care systems worldwide has raised concern among medical oncologists as to whether cancer patients are being treated and diagnosed within acceptable wait times, as recommended by guidelines. It is well established that a delay in cancer treatment has an impact on the quality of life, mental health, and clinical outcomes (such as survival and recurrence). A longer wait time is associated with a higher chance of being treated with palliative therapy over a definitive treatment. Currently, British Thoracic Society (BTS) [5], National Health Service (NHS) [6,7], RAND Corporation [8], American College of Chest Physicians [9], and Cancer Care Ontario (CCO) [10] are the only guidelines that specify recommended wait times from the date of referral to treatment in the care of cancer patients. 

Very few studies have assessed the impact of COVID-19 on waiting times in cancer patients, especially those with lung cancer. Lung cancer patients have more severe symptoms and complications of COVID-19, as it is a disease of the respiratory tract [11]. In this study we will explore some of the repercussions of the COVID-19 pandemic on lung cancer care. We will investigate the impact of the COVID-19 pandemic on the lung cancer care trajectory of patients being treated at the Peter Brojde Lung Cancer Center at the Jewish General Hospital in Montreal, Québec. The adherence to wait time guidelines will be compared before and during the COVID-19 era. In addition, lung cancer treatment pattern changes attributed to the COVID-19 pandemic will also be evaluated. 

## 2. Materials and Methods

### 2.1. Study Population

This is a retrospective cohort study including patient diagnosed with lung cancer between March 2019 and March 2021 at our center. The target population was divided into two cohorts:

- Pre-COVID-19 cohort of patients diagnosed between 1 March 2019 and 29 February 2020.
- COVID-19 cohort of patients diagnosed between 1 March 2020 and 28 February 2021.

The study was approved by the Research Ethics Board (REB). Patients were identified from the electronic health record system and included in the analysis if they had a confirmed pathological diagnosis of lung cancer, known treatment characteristics (such as dates and type of treatment), and were followed at the Jewish General Hospital. Any second opinions were excluded from the study population.

### 2.2. The Primary Objective

- To investigate the impact of the COVID-19 pandemic on lung cancer diagnoses and the lung cancer care trajectory of patients being treated at the Peter Brojde Lung Cancer Center by comparing the year 2019 to 2020.

### 2.3. Secondary Objectives

- To evaluate and compare the local wait times to the recommended guidelines before and during the COVID-19 era, and determine any repercussions of the COVID-19 pandemic on lung cancer care.
- To characterize any lung cancer treatment pattern changes attributed to the COVID-19 pandemic.

### 2.4. Data Collection

For the purpose of this study, the following information was collected from electronic medical records:

- Demographics: age, sex, smoking history;
- Diagnosis timing: referral date, date of first lung specialist consult, date of diagnosis;
- Disease characteristics: stage, histopathological diagnosis, molecular testing results;
- Treatment history: referral date, type of first definitive treatment (chemotherapy, radiotherapy, or surgery), start and end date of treatment.

### 2.5. Definitions of Wait Times

The intervals investigated for this study are shown in Table 1 with the recommended wait times from existing guidelines [9,12,13]. In this study, the wait time to be seen by a lung cancer specialist was defined as the time between referral for suspected cancer and first appointment with the lung cancer specialist. Diagnosis was defined as a date of pathological confirmation of lung cancer. The wait time to diagnosis was defined as an interval between the referral and diagnosis date. The wait time for first treatment was calculated from the date of referral and from the date of diagnosis. The decision-to-treat (DTT) to first definitive treatment (FDT) interval was defined as the interval between the date when the patient agreed to a proposed treatment plan and the date that the patient receives the first definitive treatment. The wait time for surgery was calculated as a difference between thoracic surgeon consult date and date of the surgery.

### 2.6. Statistical Analysis

In this study, the mean, median, and ranges were used to summarize patient characteristics and wait time intervals. Binary wait time variables were used to calculate the proportion of patients who met the recommended wait times. Chi-square statistics were used to define the significance of the differences. A *p*-value of less than 0.05 is considered as a significant difference. The dataset was locked on 28th February 2021. All statistical analysis was performed using the SPSS software. 

## 3. Results

**Figure 1:**
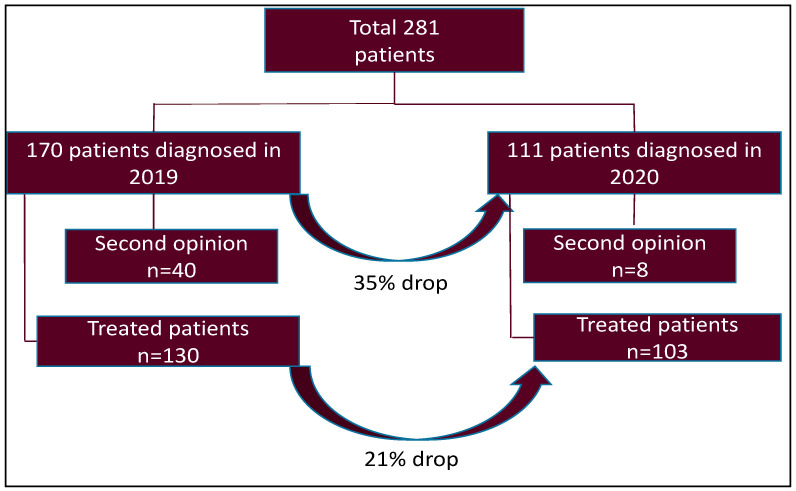
Patient flow chart.

During the study period, a total of 281 patients were diagnosed with lung cancer. This included both non-small cell lung cancer (NSCLC) and small cell lung cancer (SCLC). A total of 170 patients were included in the 2019 cohort and 111 patients in the 2020 cohort (Figure 1). The overall number of diagnosed lung cancer cases declined by 34.7% during the pandemic. After excluding all cases of second opinions, there were 130 patients diagnosed in 2019 compared to 103 patients in 2020 (Figure 1). 

Patients’ characteristics are presented in Table 2. There was no difference between the two cohorts. In both cohorts the mean age of the patients was similar, the majority were male, smokers, and were diagnosed with an advanced stage of lung cancer. 

**Figure 2:**
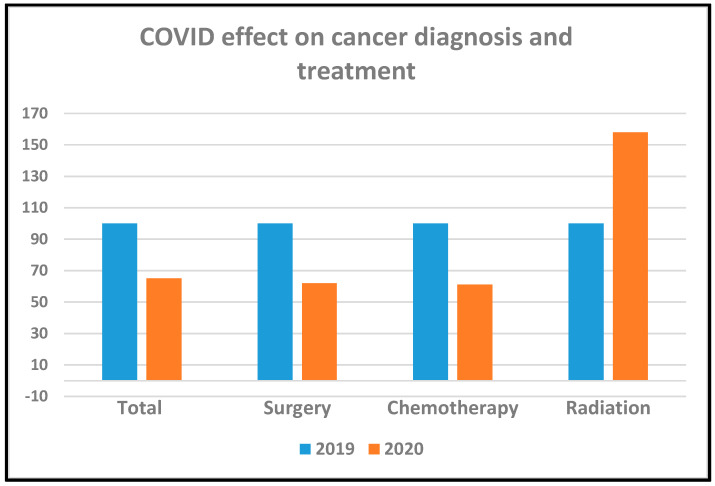
Major effect of COVID–19.

The type of treatment received is presented in Table 3. Overall, 194 patients received first definitive treatment (FDT): radiosurgery, chemotherapy, or surgery. FDT was given to 110/130 (85%) of patients in 2019 and to 84/103 (82%) of patients in 2020 (Table 2, Figure 2). Treatment patterns of early-stage disease revealed a significant increase in the utilization of radiosurgery as the first definitive treatment: 21% in 2020 vs. 7% in 2019 (*p* < 0.05) and a decrease in lung cancer surgery: 25% in 2020 vs. 38% in 2019 (*p* = 0.09). No changes were observed in systemic chemotherapy: 44/60 (73%) of patients received chemotherapy with or without immunotherapy in 2019 and 28/40 (70%) in 2020 (*p* > 0.05). The use of targeted therapy did not changed (*p* > 0.05).

### 3.1. Wait Times

Table 4 outlines the intervals and the mean wait times for the years 2019 and 2020. The percentage of patients who met the recommended wait times is also shown in Table 5. Despite the pandemic and the use of telemedicine, there was no significant delay in first appointment with a lung cancer specialist, and 72% of patients were seen within the recommended 14-day target, compared to 69% in 2019 (*p* = 0.35). We did not observe a significant delay in lung cancer diagnosis between years 2019 and 2020 (*p* = 0.15). However, the target wait time of 30 days for diagnosis was not met in 40% of patients in 2019 and 48% in 2020 (*p* = 0.29). A significant delay for lung cancer surgery was observed during the pandemic: 76 days versus 64 days in 2019 (*p* = 0.04). The most common type of surgery was VATS lobectomy: 27/46 (58%) in 2019 and 16/26 (62%) in 2020; followed by wedge resection: 18 (38%) in 2019) vs. 10 (38%) in 2020. One patient had a pneumonectomy in 2019. No statistical difference was observed. 

Mean wait time for definitive radiation was similar before and during the pandemic: 35 vs. 46 days, respectively. The majority of patients were started on radiation therapy within the recommended wait time of 42 days (79% in 2019 and 71% in 2020). Although there was no significant differences in the mean wait times for the remaining intervals, less than 50% of patients met the target wait times in both cohorts (Table 4 and Table 5).

Among the patients who received systemic chemotherapy, 37% in 2019 and 35% in 2020 were treated within the 28-day target (Table 5). However, for targeted therapy, the proportions of those treated within 28 days were 64% in 2019 and 66% in 2020.

### 3.2. Molecular Testing

The molecular tests used in our institution included next-generation sequencing (NGS), liquid biopsy, and NanoString (Table 6). In both cohorts, NGS was the most common molecular test with a mean wait time of 15 days in 2019 compared to 18 days in 2020 (*p* = 0.03). There was no significant delay in getting liquid biopsy or NanoString results between the two cohorts.

## 4. Discussion

In this retrospective chart review study comparing the lung cancer trajectory before and during the COVID-19 pandemic, we found that referral to a lung cancer specialist and subsequent diagnosis of lung cancer declined by 34.7% during the pandemic. Fear related to contracting COVID-19, quarantining, and stay-at-home orders have caused patients to be more apprehensive to seek care for emergent issues [2]. This finding is further supported by the study conducted by Dr. Reyes and colleagues that found a 38% decrease in new lung cancer cases in 2020 compared to the pre-COVID-19 era [14]. According to Quebec Ministry of Health, about 4100 people may have gone undiagnosed with all cancer types during the pandemic. 

Despite the pandemic, we were able to deliver definitive treatment (FDT) to the same number of patients (85% in 2019 vs. 82% in 2020). The majority of patients in our study were seen by a lung cancer specialist within a target wait time of 14 days (72%). This has not significantly changed when compared to the pre-COVID era. We found no difference in wait time to obtain a lung cancer diagnosis before and during the pandemic: 40% and 48% of patients with suspected lung cancer had the diagnosis confirmed within the target of 30 days, respectively. We observed a small delay for next-generation sequencing test results. The question remains if these delays changed the outcomes. Dr. Bissonette and colleagues have reported that in patients who had diagnostic and planning PET-CT 21 days apart, the overall upstaging occurred in 25% of patients. The rate of overall upstaging increased with longer delays between staging and treatment planning PET-CT scans [15].

The COVID-19 pandemic revealed the needs for more information and guidance for changes in existing practice among lung cancer specialists including thoracic surgeons [16]. Many institutions have changed the treatment plan in order to minimize the risk of patient exposure, in accordance with recommendations of certain expert groups [17,18,19,20,21,22]. The consensus was that select treatment is still possible in a 28-day period. However, in this present crisis, lung cancer surgeries should be deferred if possible in patients with a low risk of progression [22]. Furthermore, considering the availability of certain treatments, alternatives should be considered. The Quebec Lung Cancer Network recommended stereotactic radiation as an alternative to surgery for stage I–IIa lung cancers [17]. As a result of prioritization of available treatments, we observed a 14% (21% in 2020 vs. 7% in 2019) increase in radiosurgery given with the curative intent to early-stage disease and a 13% (25% in 2020 vs. 38% in 2019) decrease in surgical resections during the pandemic. Overall, in the province of Quebec an 18% decrease in lung cancer surgeries was observed during the pandemic compared to 2019 [3]. This decline in surgery was partially due to reduced operating room hours, the lack of medical staff, and long waiting list. When radiosurgery was offered as an alternative to surgery, some patients preferred to receive radiosurgery rather than surgery, to minimize their hospital stay and decrease their risk of getting COVID-19 infection. In our study, 70% of patients exceeded the 28-day target wait time for lung cancer surgery. According to the Quebec Ministry’s report, very few patients had a cancer surgery within 0–56 days, while a greater proportion of cases were waiting for surgery more than 56 days. Participants of the Spanish Thoracic Society study also reported a decrease in surgical resection and prolonged wait time for surgery with a mean time exciding one month [20]. The majority of our patients receiving radiation therapy were treated within the recommended wait time. This observation is similar to the one reported by Nadpara et al., who concluded that wait times from diagnosis to first radiotherapy was shorter than the wait time for surgery [23]. Rapid diagnostic assessment programs (DAPs) and enhanced recovery protocols (ERPs) may improve timeliness of surgical care [3]. Dr. Hubert and colleagues reported that using the DAP, the median time between the patient’s first clinic visit and referral to surgery was 30 days, and the median time between surgical consult and treatment was 29 days [24]. To date, there is no consensus whether a longer preoperative delay has a negative effect on overall survival. Yang and colleagues reported that patients who had surgery 38 days or more after diagnosis had a significantly worse 5-year overall survival than patients who had surgery earlier (hazard ratio (HR) 1.13; 95% CI, 1.02–1.25; *p* = 0.02). Conversely, Quarterman and colleagues reported a median interval between presentation and surgical treatment of 82 days. They were unable to demonstrate a negative effect of longer preoperative delays on overall survival (*p* = 0.54) [25].

The type of chemotherapy received did not vary before and during the COVID-19 pandemic. However, less than 40% of our patients met the target of 28 days before and during the pandemic for systemic chemotherapy, and more than 60% of patients met the target for targeted therapy. Hospital staff shortages and increased workload of those who continued to work in cancer care and patients recurrent visits to the hospital might explain the delay of systemic chemotherapy. Fujita and colleagues reported delay in systemic chemotherapy compared to targeted treatment in lung cancer patients. They also found that adding immunotherapy to standard chemotherapy causes a longer delay during the COVID-19 era [26].

## 5. Limitations

This study is, to our knowledge, the largest single-institution report comparing lung cancer trajectory before and during the COVID-19 pandemic in the province of Quebec. There are, however, limitations to our study. This is a retrospective chart review from a single institution. The study population is relatively small and is not representative of the general population and prone to selection bias. The results may not be generalizable to other institutions.

## 6. Conclusions

COVID-19 seems to have had a major impact at our lung cancer center for the diagnoses and treatment patterns of our lung cancer patients. Diagnoses of lung cancer dropped off significantly during the pandemic. Many oncologists fear that they will see an increase in newly diagnosed lung cancer patients in the coming year, as vaccination rates continue to increase. In addition, treatment patterns seemed to indicate a decrease in surgery and an increase in radiosurgery. This study is still ongoing and further data will be collected and analyzed to better understand the total impact of the COVID-19 pandemic on our lung cancer patient population.

## Figures and Tables

**Table 1:** Recommended wait times for lung cancer patients.

Wait Time	Mean Time (Days)	Guidelines
Referral → Lung cancer specialist	14	National Health Service [13]
Referral → Diagnosis	30	British Thoracic Society [12]
Referral → First treatment	62	National Health Service
Diagnosis → First treatment	30	British Thoracic Society
Decision-to-treat → First definitive treatment	31	British Thoracic Society
Diagnosis → First chemotherapy	28	British Thoracic Society
Surgery consult → Surgery	28	British Thoracic Society
Radiation consult → First radiation therapy	42	RAND Corporation [9]

**Table 2:** Patients’ characteristics.

Characteristics		2019 *n* = 130	2020 *n* = 103
Age (mean; range)		70 (40–96)	71 (42–92)
Sex (male/female)		73/57	56/47
Cancer stage (*n*/%)	Early stage (T_1–3_N_0–1_M0) Locoregional (T_1–4_N_2–3_M0) Advanced/metastatic stage (T_any_N_any_M_1_)	42 (32) 20 (15) 68 (53)	33 (35) 11 (10) 59 (55)
Smoking history (*n*/%)	Former/current smoker Non-smoker	99 (76) ^1^ 31 (24)	74 (74) 29 (26)
Treatment type: (*n*/%)	FDT ^2^ PT ^3^	110 (85) 20 (15)	84 (82) 19 (18)

^1^ = data from two patients are missing, ^2^ = first definitive treatment, ^3^ = palliative treatment.

**Table 3:** Type of first treatment.

Type of First Treatment		2019 *n* = 110	2020 *n* = 84	*p*-Value
Radiosurgery (*n*/%)		8 (7)	18 (21)	<0.05
Chemotherapy (*n*/%):	Total Standard systemic chemotherapy Immunotherapy ± chemotherapy Targeted therapy	60 (54) 21 (37) 23 (36) 17 (27)	40 (47) 12 (30) 16 (40) 12 (30)	0.07
Surgery		42 (38)	26 (25)	0.09

**Table 4:** Mean wait times before (2019) and during COVID-19 era (2020).

Interval	Recommended Wait Times (days)	2019 *n* = 130		2020 *n* = 103		*p*-Value
		*n*	Mean (SD ^1^) Days	*n*	Mean (SD ^1^) Days	
Referral → Lung cancer specialist	14	130	12 (14)	103	11 (13)	0.67
Referral → Diagnosis	30	130	59 (51)	103	59 (67)	0.94
Referral → First treatment	62	130	79 (47)	103	82 (71)	0.76
Wait for path report	-	130	8 (7.3)	103	8 (7.6)	0.98
Decision-to-treat to FDT ^2^	31	130	52 (48)	103	51 (61)	0.94
Diagnosis to chemotherapy	28	64	38 (25)	39	34 (24)	0.95
Diagnosis to RT ^3^	42	24	35 (30)	38	46 (33)	0.31
Surgical consult to surgery	28	42	64 (43)	27	76 (83)	0.04
Wait for molecular test results	7	73	21.9 (9.9)	51	21.6 (8.7)	0.90

^1^ Standard deviation, ^2^ first definitive treatments, ^3^ radiation treatment.

**Table 5:** Meeting the wait time standards.

Interval	Recommended Wait Time	2019	2020	*p*-Value
		Proportion (%)		
Referral → Lung cancer specialist	14 days	90/130 (69)	74/103 (72)	0.35
Referral → Diagnosis	30 days	52/130 (40)	49/103 (48)	0.15
Referral → First treatment	62 days	56/130 (43)	49/103 (48)	0.29
Diagnosis → First systemic chemotherapy ^1^	28 days	24/64 (37)	7/20 (35)	0.45
Diagnosis → First targeted chemotherapy	28 days	11/17 (64)	5/7 (66)	0.43
Surgical consult → Surgery	28 days	6/42 (14)	8/26 (30)	0.12
Radiation oncology consult → Radiation treatment	42 days	17/24 (71)	30/38 (79)	0.52

^1^ Systemic treatment includes chemotherapy, IO, or combination of the two.

**Table 6:** Mean wait time for molecular testing.

Type of Molecular Test	2019 *n* = 127		2020 *n* = 106		*p*-Value
	*n*	Mean (SD ^1^) Days	*n*	Mean (SD ^1^) Days	
NGS ^2^	66	15 (6)	62	18 (9)	0.03
Liquid biopsy	9	6 (10)	15	4 (18)	0.81
NanoString	18	25 (11)	19	27 (18)	0.50
Total	100	16 (9)	96	18 (14)	0.11

^1^ Standard deviation, ^2^ next-generation sequencing.

## Data Availability

The data are not publicly available due to privacy issue.
